# Integrated bioinformatics identifies key mediators in cytokine storm and tissue remodeling during *Vibrio mimicus* infection in yellow catfish *(Pelteobagrus fulvidraco)*

**DOI:** 10.3389/fimmu.2023.1172849

**Published:** 2023-05-22

**Authors:** Yang Feng, Jiao Wang, Wei Fan, Yi Geng, Xiaoli Huang, Ping Ouyang, Defang Chen, Hongrui Guo, Huidan Deng, Weimin Lai, Zhicai Zuo

**Affiliations:** ^1^ College of Veterinary Medicine, Sichuan Agricultural University, Chengdu, Sichuan, China; ^2^ Department of Aquaculture, College of Animal Science & Technology, Sichuan Agricultural University, Chengdu, Sichuan, China

**Keywords:** *Vibrio mimicus*, Yellow catfish (*Pelteobagrus fulvidraco*), skin and muscle, histopathology, integrated bioinformatics, signaling pathway

## Abstract

**Introduction:**

The pathogenesis of *Vibrio mimicus* infection in yellow catfish (*Pelteobagrus fulvidraco*) remains poorly understood, particularly regarding the impact of infection with the pathogen on primary target organs such as the skin and muscle.

**Methods:**

In this study, we aim to analyze the pathological intricacies of the skin and muscle of yellow catfish after being infected with *V. mimicus* using a 1/10 LC_50_ seven-day post-infection model. Furthermore, we have utilized integrated bioinformatics to comprehensively elucidate the regulatory mechanisms and identify the key regulatory genes implicated in this phenomenon.

**Results:**

Our histopathological examination revealed significant pathological changes in the skin and muscle, characterized by necrosis and inflammation. Moreover, tissue remodeling occurred, with perimysium degeneration and lesion invasion into the muscle along the endomysium, accompanied by a transformation of type I collagen into a mixture of type I and type III collagens in the perimysium and muscle bundles. Our eukaryotic transcriptomic and 4D label-free analyses demonstrated a predominantly immune pathway response in both the skin and muscle, with downregulation observed in several cell signaling pathways that focused on focal adhesion-dominated cell signaling pathways. The upregulated genes included *interleukins* (IL)-1 and -6, *chemokines*, and *matrix metallopeptidases* (*mmp*)-9 and -13, while several genes were significantly downregulated, including *col1a* and col1a1a. Further analysis revealed that these pathways were differentially regulated, with *mmp*-9 and *mmp*-13 acting as the potential core regulators of cytokine and tissue remodeling pathways. Upregulation of *NF-κB1* and *FOSL-1* induced by *IL-17C* and *Nox 1/2*-based NADPH oxidase may have held matrix metallopeptidase and cytokine-related genes. Also, we confirmed these relevant regulatory pathways by qPCR and ELISA in expanded samples.

**Discussion:**

Our findings unequivocally illustrate the occurrence of a cytokine storm and tissue remodeling, mediated by interleukins, chemokines, and MMPs, in the surface of yellow catfish infected with *V. mimicus*. Additionally, we unveil the potential bidirectional regulatory role of MMP-9 and MMP-13. These results provide novel perspectives on the intricate immune response to *V. mimicus* infection in yellow catfish and highlight potential targets for developing therapies.

## 1 Introduction

Vibriosis, which is caused by members of the genus *Vibrio*, is one of the most prevalent fish diseases and a common cause of foodborne illness and wound infections (1). Among others, *Vibrio mimicus* is a highly pathogenic species that infects many aquatic and terrestrial animals, including humans. *Vibrio mimicus* is associated with several dysfunctions and pathologies in humans such as gastroenteritis, wound infections, and septicemia (2). *Vibrio mimicus*, which is widely distributed in both freshwater and estuarine environments, is a virulent pathogen causing septicemia, resulting in high morbidity and mortality in farmed fish. Various species are susceptible to infection caused by *V. mimicus*, including members belonging to Perciformes (3), Cypriniformes (4), and Siluriformes (5), as well as crustaceans such as *Procambarus clarkia* (6) and *Eriocheir sinensis* (7). Several Siluriformes species are widely cultured in China, such as southern catfish (*Silurus meridionalis*), yellow catfish (*Pelteobagrus fulvidraco*), and channel catfish (*Ictalurus punctatus*), all of which are highly susceptible to *V. mimicus* (5, 8). Onset of clinical signs in infected fish is typically after 5-7 days post-infection, and the mortality rate is more than 90% (5). Pathological lesions related to infection of catfish with *V. mimicus* have been previously reported. These include focal hemorrhage, muscular necrosis, and inflammation in various organs, such as the heart and muscles (5). Although the infection is believed to trigger a complex immune response that activates inflammation, the underlying molecular mechanisms are largely unknown and warrant further studies.

Yellow catfish is a valuable freshwater fish which is widely cultured in China for its excellent meat quality and high market value. The annual production of yellow catfish is approximately 500,000 tons in China (9), and the economic value of the total harvest of the fish exceeds four billion dollars. However, *V. mimicus* infection is a serious challenge for sustainability of catfish production and results in substantial economic losses to fish farmers. Understanding the mechanisms associated with infection with *V. mimicus* and developing more effective treatments against the pathogen can improve health status and welfare of farmed catfish (10). Currently, a significant literature exists on the virulence factors of *V. mimicus* (11, 12), including our prior research which identified several factors such as the type II secretion system, TonB iron uptake system, hemolysin, sialic acid, and prtV metalloprotease, that contribute to its pathogenesis (13–16). Additionally, Lee et al. discovered the toxic effects of *V. mimicus* phospholipase on fish cells (17). However, the understanding of the host specificity mechanisms of *V. mimicus* remains limited. Only Tao et al. have studied the effect of *V. mimicus* on cell catalytic activity, immunity, antioxidant response, and cytoskeleton formation in infected carp (18), while Li et al. have investigated the mucosal immune regulatory pathways of the grass carp intestine post-infection with *V. mimicus* (19). Despite the fact that skin and muscle are the main target organ of *V. mimicus* (20), there exists a paucity of studies pertaining to mechanisms associated with host response to infection with the pathogen. This study aims to comprehensively analyze integrated bioinformatics to uncover the mechanisms involved in infection with *V. mimicus* in yellow catfish, providing novel insights into the complex immune response to *V. mimicus* infection and identifying new targets for vaccine and therapy development.

## 2 Materials and methods

### 2.1 *V. mimicus* infected model

The strain *V. mimicus* SCCF01, isolated from diseased yellow catfish at a commercial aquaculture site in Sichuan province, China, in 2014, was identified using genome analysis (21). For the challenge trial, *V. mimicus* strains were incubated in BHI at 28°C for 24 hours. Yellow catfish (69.6 ± 6.8 g) were procured from a commercial farm in Wenjiang, Sichuan, China, and acclimatized at the research facility for two weeks. They were fed commercial pellets twice a weekday and maintained under a light: dark cycle of 12:12 h. Water temperature, water ammoniacal nitrogen and nitrite, and water pH were maintained at 23 ± 2°C, 0-0.02 mg/L, at 23 ± 2°C, and 7.0-7.5, respectively. The culture water was replaced daily after pretreatment with an aeration process. All animal handling procedures were approved by the Animal Care and Use Committee of Sichuan Agricultural University and followed the guidelines for animal experiments of Sichuan Agricultural University under permit number 2020103001.

Healthy yellow catfish with good vitality, complete appendages, and no disability were randomly allocated to control and challenge groups. The control group was treated with 0.65% physiological saline, while the challenge group was treated with purified strains (1.0 × 10^4^ CFU/mL). The fish were immersed in different pre-trialed water for 30 minutes and trans-cultured in fresh water. During the experiment, the water temperature was maintained at 23 ± 2°C, the pH was 7.5-8.5, and commercial feed for yellow catfish was fed at 5% fish weight once daily at 6:00-7:00 pm. The experiment duration was seven days, and the mortality and activity of the fish in each group were observed and recorded. At seven days post-challenge, yellow catfish skin, and muscle tissues were collected randomly from each group, fixed using Neutral Buffered Formalin Fixative for paraffin sectioning, and frozen in liquid nitrogen for RNA and protein analysis and storage at -80°C.

### 2.2 Light microscopy

The skin and muscle tissues were fixed in a fixative, trimmed into cassettes, dehydrated in graded ethanol solutions, cleared in xylene, and embedded in paraffin wax. Subsequently, four μm sections were prepared for microscopic analysis using hematoxylin-eosin (H&E), Alcian Blue/Periodic Acid-Schiff (AB-PAS), Masson Trichrome, and Picrosirius Red staining. The degree of histopathological changes in the organs, including swelling, necrosis, and inflammation, were scored based on the method proposed by Baums et al. (22). A histopathological score ranging from 0 to 6 was assigned depending on the degree and extent of alteration, where 0 indicated no change, 2 indicated mild occurrence, 4 indicated moderate occurrence, and 6 indicated severe occurrence (diffuse lesion), with intermediate values also considered. The histopathological scores were assessed by a pathologist in a blinded manner.

### 2.3 Eukaryotic transcriptome analysis

#### 2.3.1 RNA library construction and sequencing

RNA-seq was performed by separately sampling four skins and muscles from each group. The concentration and purity of total RNA extracted were measured using a Nanodrop, and its integrity was assessed using Agilent 2100. Magnetic beads with Oligo (dT) were used to enrich mRNA, which was then fragmented using a fragmentation buffer. cDNA was synthesized using the mRNA template and purified with AMPure XP beads. The ends were repaired, adapters were connected, and AMPure XP beads were used for fragment size selection. The library was subjected to PCR amplification, and PCR products were purified using AMPure XP beads to obtain the final library, which was then diluted to 1.5 ng/μL. The effective concentration of the library was quantified using q-PCR (the effective concentration >2nM), and transcriptome sequencing was performed using Illumina HiSeqTM 2500 based on the effective concentration.

#### 2.3.2 Differential miRNA expression analysis

The raw data quality was evaluated, and the unigenes were obtained by assembling the reads and removing redundancy. Clean reads were obtained by removing subassembly and low-quality sequences, and transcripts were performed by merging the clean reads using Trinity 0.12.8 (23). Then, clean reads were compared with the referred *P. fulvidraco* genome GCF_022655615.1 (https://www.ncbi.nlm.nih.gov/assembly/GCF_022655615.1/) using BOWTIE to obtain the SAM/BAM file. The list of read counts was obtained by clustering and quantifying the transcripts using CORSET 1.03. Fragments from each sample were compared to transcripts using BOWTIE, and the abundance of information for each fragment was statistically analyzed. Annotation information was obtained by comparing the unigenes with the Ref-Seq non-redundant proteins (Nr), Swiss-prot, Pfam, complete eukaryotic genomes (KOG), Gene Ontology (GO) analysis, and Kyoto Encyclopedia of Genes and Genomes (KEGG) databases (e value ≤ 1e-5) using BLAST software. Transcripts Per Million (TPM) values were used to determine the expression levels of differentially expressed genes (DEGs). DESeq2 algorithms were used to select a subset of DEGs, and the P-value was corrected by multiple hypothesis tests using the BH method (screening threshold: FDR ≤ 0.05, Abs (log2 Fold Change) ≥ 2). The annotation information was used to screen for DEGs, and KEGG significant enrichment analysis was performed on the DEGs to determine regulatory pathways.

### 2.4 4D label-free quantitative proteomic analysis

Proteomic profiling was performed on three skins from each group using liquid chromatography-tandem mass spectrometry (LC-MS) analysis. The skins were lysed in a lysis buffer containing 8 M urea and 1% protease inhibitor cocktail using ultrasonic waves and then centrifuged at 12,000 g for 30 min. The supernatant was collected, and protein concentration was determined using the bicinchoninic acid assay kit. Dithiothreitol (DTT) was added to the protein solution to a final concentration of 5 mM, followed by a reduction reaction at 56°C for 30 min. Next, iodoacetamide was added to a final concentration of 11 mM and incubated for 15 min at room temperature in the dark. The urea was diluted by supplementing triethylammonium bicarbonate to a concentration of less than 2 M. Trypsin was added to the protein solution at a ratio of 1:50 (protease: protein, m/m) for overnight hydrolysis, followed by a ratio of 1:100 (protease: protein, m/m) for another four hours. The resulting peptides were separated using a Brucker NanoElute ultra-high-performance liquid chromatography (UHPLC) system with a liquid phase gradient setting of 0–45 min, 2%-22%B; 45–50 min, 22%-35%B; 50–55 min, 35%-80%B; 55–60 min, 80%B, and a flow rate maintained at 300 nL/min. The peptides were ionized using a Capillary ion source and analyzed by timsTOF Pro mass spectrometry with parallel accumulated serial fragmentation (PASEF) mode. The data were processed using the Proteome Discoverer™ Software v2.2 against the *P. fulvidraco* proteome database (Genebank: GCF_022655615.1_HZAU_PFXX_2.0_protein.faa_unique.fasta), and differential proteins were identified using the student’s *t-test* with a fold change of ≥1.20 or ≤0.83 and *P* value < 0.05. Perseus Version 1.6.15.0 was used for statistical analysis, and a heatmap was plotted using k-means clustering. Protein GO analysis and KEGG pathway enrichment analysis were performed to examine the biological pathways of the differentially expressed proteins. The enrichment analysis was also performed using Metascape software. The statistical significance was determined using a corrected *P* value < 0.05, indicated by the Benjamini-Hochberg method. Correlation analysis was performed using the STATS Package and pcaPP package of the R language.

### 2.5 Real-time quantitative polymerase chain reaction

RNA isolation from the skin and muscle was performed using an animal tissue total RNA extraction kit (Fuji, Chengdu, China). Subsequently, cDNA was synthesized from 1 μg of RNA using a PrimeScript RT reagent kit with gDNA Eraser (Fuji) and a Thermo Cycler (BioRad, Hercules, CA, USA) were used to perform qPCR, where *18S*, *β-actin*, and *GAPDH* were employed as reference genes to determine the relative expression of target genes (24). The primer sequences of each gene used in this analysis are given in **Table S1**. The ten μL reaction mixture for qPCR contained five μL SYBR Green PCR Master Mix, three μL diethylpyrocarbonate-treated water, 0.5 μL of forward primer, 0.5 μL of reverse primer, and one μL cDNA. The following thermal cycling program was used: 30 s at 95°C for one cycle, 40 cycles of amplification at 95°C for 10 s, a melting temperature based on the specific primer pair for 20 s, and 72°C for 10 s. A melting curve analysis was conducted at the end of each run to distinguish between specific and nonspecific reaction products. The 2−ΔΔCT method was used to calculate relative changes in mRNA transcript expression from the qPCR results, where ΔCT = CT_target gene_ – CT_references genes_, and ΔΔCT = ΔCT_experimental_ - ΔCT_control_ (25).

### 2.6 Enzyme-linked immune response

The tissue weight was measured, and a volume of ethanol nine times that of the weight (g) was added (volume (mL) = 1:9). The sample was mechanically homogenized on ice, followed by centrifugation at 600 G for 10 minutes. The activity of MMP-9, Type I Collagen, TLR 5, IL-1β, and IL-6 was determined using fish-specific kits (MIBIO, Shanghai, China), according to the manufacturer’s instructions.

### 2.7 Statistical analysis

The mean and standard deviation were used to express the results, and statistical significance was assessed by performing Student’s t-test using SPSS software version 20.0 (IBM Corp., Armonk, New York, USA). A P-value of less than 0.05 was considered significant, while a P-value of less than 0.01 was considered highly significant.

## 3 Results

### 3.1 Histopathological analysis of skin and muscle changes in yellow catfish infected with *V mimicus*

**Figure 1:**
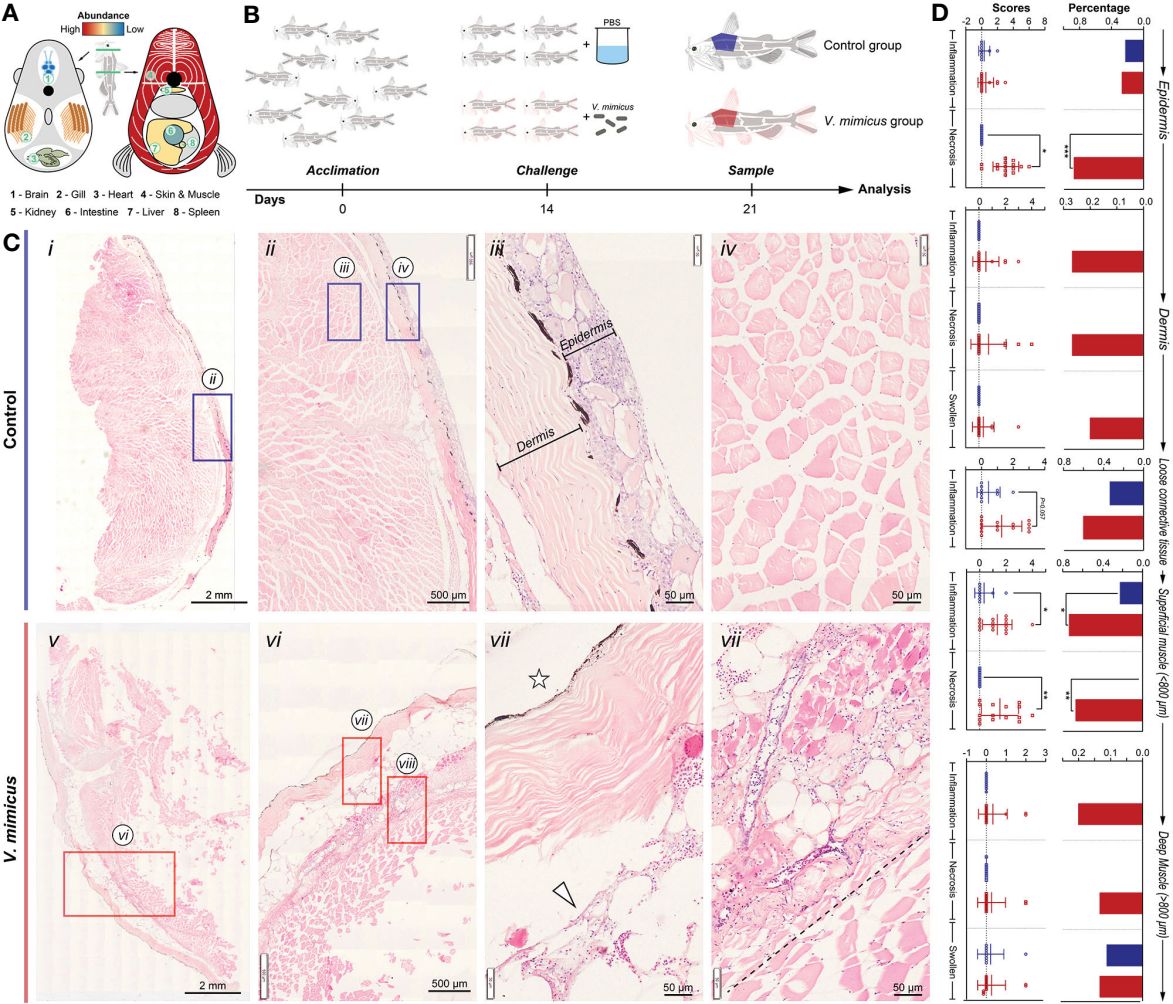
Histopathological evaluation of skin and muscle in yellow catfish. **(A)** Schematic representation of *V. mimicus* parasitizing yellow catfish (20); **(B)** Experimental challenge with *V. mimicus* in catfish; **(C)** Histopathology of skin and muscle in control and *V. mimicus*-infected groups; **(D)** Histopathological scores of skin and muscle in control and *V. mimicus*-infected groups. N _control_=9, n*_v. mimicus_* =15. ∗ P < 0.05, ∗∗ P < 0.01, *** P < 0.001 represents a significant or highly significant difference between groups.

In our previous study, skin and muscle were identified as target organs after *V. mimicus* infection in yellow catfish (**Figure 1A**). This study aimed to investigate the related pathological changes and response mechanisms of skin and muscle using a post-infection model of 1/10 LC_50_ for seven days (**Figure 1B**). Under histopathology, the skin and muscle of the control group exhibited neatly arranged layers of homogenous stained structures with loose connective tissue (subcutaneous layer) connecting the skin and muscle (**Figures 1C** i-iv). However, after *V. mimicus* infection, the skin and muscle displayed significant pathological changes, including necroinflammation (**Figures 1C** v-vii). The epidermis demonstrated varying degrees of structural alterations, such as mucinous cell proliferation, multinucleated mucinous cells, epithelial cell chemosis, uneven epidermal thickness, neutrophil infiltration, and even overall detachment. The dermis exhibited a disorganized fibrous structure, edema-like laxity, and neutrophil infiltration. Neutrophil infiltration was evident in the loose connective tissue, while sarcoplasmic coagulation, myofibrillar necrosis, and neutrophil infiltration were found in the muscle layer. Additionally, varying degrees of vasculitis was observed in the skin and muscle layers. The lesions of interest in both groups were scored and analyzed statistically according to the lesions. During the evaluation process, skin and muscle were divided into five layers: epidermis, dermis, loose connective layer, superficial muscle layer (within ≈800 μm from the loose layer), and deep muscle layer (outside ≈800 μm from the loose layer). Based on the analysis of the five-layer structure, cell necrosis changes displayed an outward-to-inward progression based on severity. In contrast, inflammatory changes primarily developed in the loose connective layer and superficial muscle layer based on severity (**Figure 1D**).

### 3.2 Tissue remodeling in perimysium and foci of the muscle bundles

**Figure 2:**
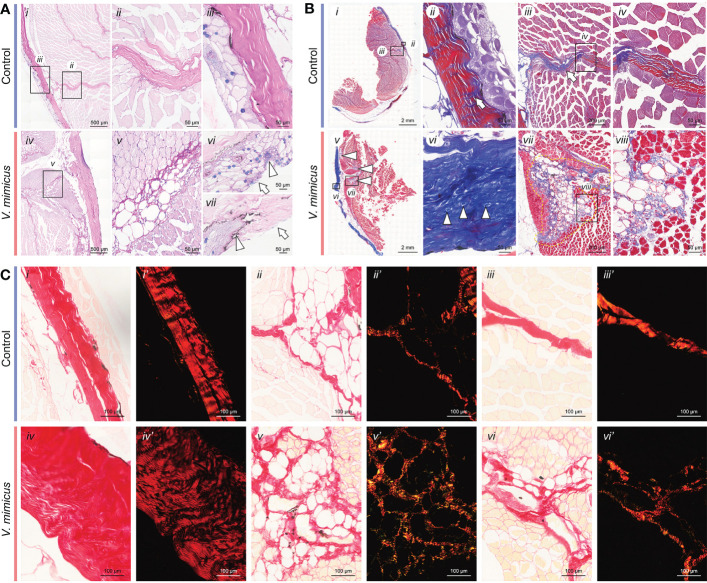
Staining of skin and muscle in yellow catfish. **(A)** AB-PAS staining of skin and muscle; **(B)** Masson staining of skin and muscle; **(C)** Picrosirius Red staining of skin and muscle. n _control_=9, n*_v. mimicus_* =15.

In our study, AB-PAS staining was applied to the control samples, showing a blue-purple coloration of the epidermis, purplish-red dermis, and pink muscle fibers (**Figures 2A** *i-iii*). However, after *V. mimicus* infection, the perimysium exhibited vacuolar degeneration, and the lesion extended to myofibers with muscle bundles, accompanied by cellular infiltrates within the perimysium (**Figures 2A** *iv-vii*). Masson staining revealed a reduction in red-stained portions of the dermis after *V. mimicus* infection, along with the appearance of small body-like structures (**Figures 2B**). Furthermore, the lesion entered the muscle along the endomysium. Only the vacuolated designs of the endomysium were found to be atrophied, with the proliferation of collagen fibers within (**Figures 2B** *vii-viii*). Picrosirius Red staining was employed to differentiate the type of collagen, showing red in the perimysium and pink in the epidermis and myofibrils of the control sample (**Figures 2C** *i-iii*). Collagen under polarized light exhibited different refractive lights. The dermis, reticular structures of the lesioned areas within the muscle, and perimysium were bright red, and a dark background was observed in the epidermis and myofibers (**Figures 2C** *i’-iii’*). However, collagen in the dermis of the *V. mimicus* group appeared sparse and dark red under polarized light, and the reticular structures in the intramuscular lesion area showed different colors other than red (**Figures 2C** *iv-vi’*). Upon magnification, collagen structures, mainly type I collagen, were observed in the skin and muscles of the control group. In contrast, a mixed state of type I and type III collagen was found in the muscle of the *V. mimicus* group. Our analysis revealed significant tissue remodeling in reticular structures and foci of the muscle bundles, indicating the extent of damage caused by the infection.

### 3.3 Bioinformatics analysis shows increased immune response and decreased structural stability

**Figure 3:**
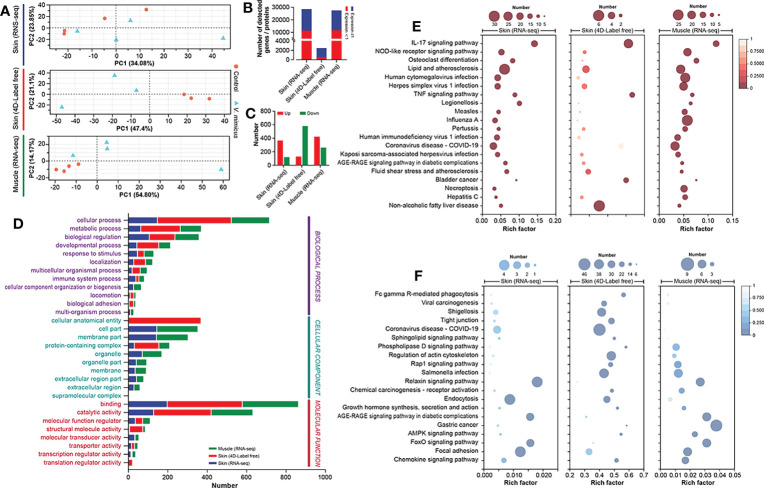
Integrated bioinformatics analysis of skin and muscle. **(A)** PCA analysis of samples (Skin transcriptome: n=4, Skin 4D Label-free: n=3, and Muscle transcriptome: n=4); **(B)** Number of genes expressed in the three bioinformatics methods; **(C)** Differential gene expression statistics for the three bioinformatics methods; **(D)** GO annotation analysis of genes; **(E)** KEGG enrichment analysis of upregulated genes; **(F)** KEGG enrichment analysis of downregulated genes. Comparison between the *V. mimicus* group and the control group.

In this study, we performed bioinformatics analyses of the skin and muscle tissues using the following strategies: 1) identification of shared regulatory pathways between the two organs through transcriptome analysis, and 2) clarification of gene-protein regulatory pathways in the skin through transcriptome and 4D Label-free analyses. The PCA analysis of the three bioinformatics approaches is presented in **Figure 3A** after quality control processing of the data (**Table S2**; **Figure S1**). In this study, a total of 28,444, 2,435, and 27,427 expressed genes/proteins were identified from the skin transcriptome, skin 4D Label-free, and muscle transcriptome, respectively (**Table S3**; **Figure S2**). Among them, 17,187, 1,744, and 15,830 genes/proteins were found to be expressed at a minimum of 1 peptide number of proteins or 1 transcript per million of genes (**Figure 3B**). Using the DESeq2 algorithm with a screening threshold of: |log2FC| >=1 & *P* adjust < 0.05, we performed differential gene analysis between groups and obtained a total of 488 (up: 367; down: 121), 707 (up: 128; down: 579), and 682 (up: 422; down: 260) differentially expressed genes/proteins from the three bioinformatics analyses (**Figure 3C**). The GO annotation analysis of the differentially expressed genes/proteins revealed that cellular processes, binding, and hydrolytic activities were significantly enriched in all three bioinformatics analyses (**Figure 3D**). The Directed Acyclic Graph (DAG) analysis of GO enrichment of the transcriptome also indicated an acute inflammatory response in the skin and muscle, as well as changes in purine metabolism and extracellular matrix (**Figure S3**). The KEGG enrichment analysis showed that the most enriched pathways in the three bioinformatics analyses were the interleukins (IL) -17 signaling pathway, Ribosome, and IL-17 signaling pathway, respectively (**Figure S4**). The integrated analysis of the results demonstrated that the upregulated expression genes were mainly associated with inflammatory pathways dominated by the IL-17 signaling pathway (**Figure 3E**). In contrast, the downregulated pathways were mainly focal adhesion-dominated cell signaling pathways (**Figure 3F**). Our findings suggest that the skin and muscle tissues exhibited a significantly increased immune response and decreased structural stability.

### 3.4 MMP-9&-13 mediate inflammation and tissue remodeling

**Figure 4:**
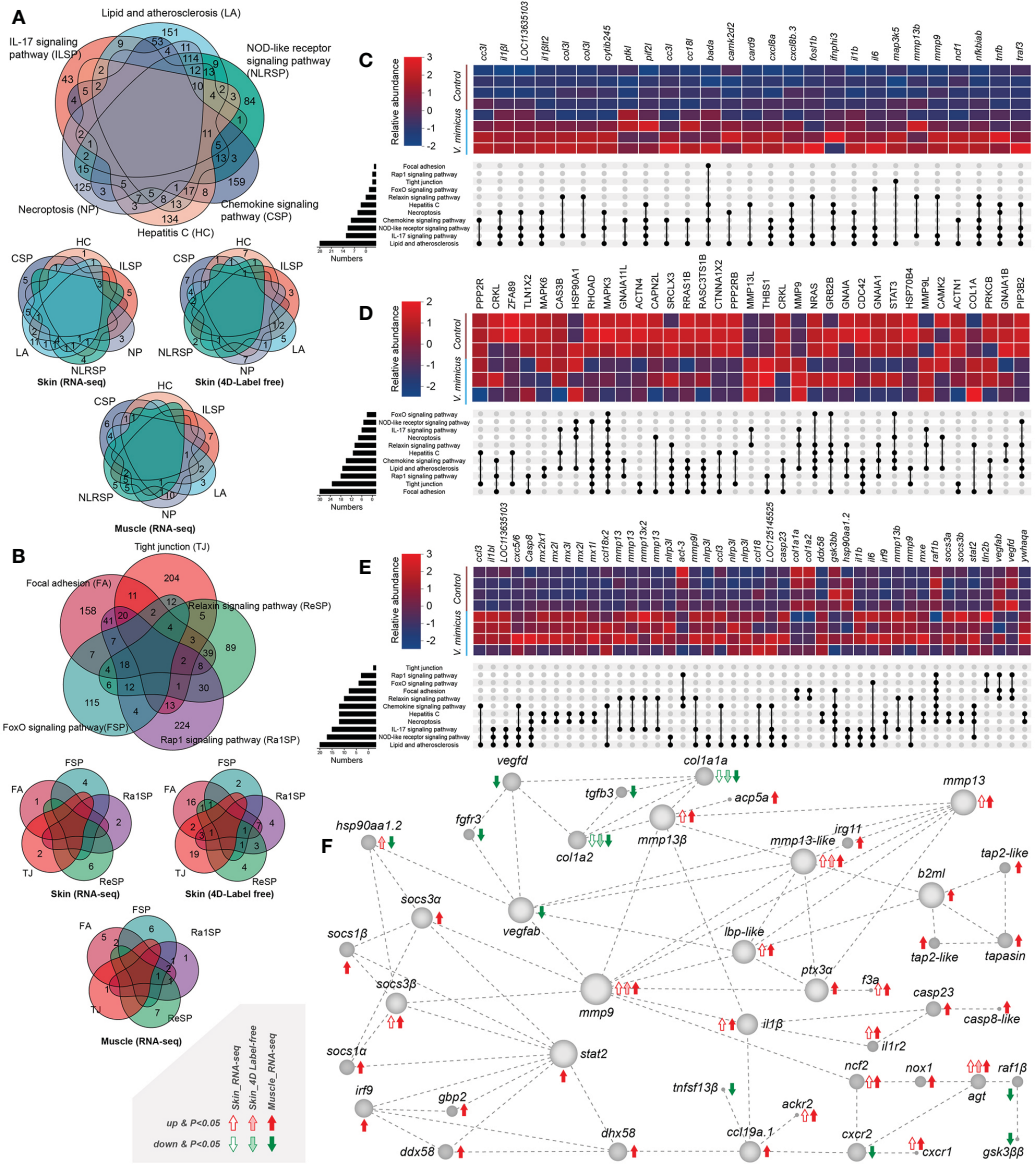
Gene expression in enriched pathways. **(A)** Venn diagram of genes in significant enrichment pathways of upregulated genes; **(B)** Venn diagram of genes in critical pathways of downregulated genes; **(C)** Expression of significant genes in the skin transcriptome; **(D)** Relative expression of major proteins in the skin proteome; **(E)** Expression of significant genes in the muscle transcriptome; **(F)** Diagram of the major protein interaction network.

We performed KEGG enrichment analysis to identify up-regulated or down-regulated pathways with high enrichment and significance. Among these pathways, we found that many genes were shared between up-regulated and down-regulated pathways and between the skin transcriptome, skin proteome, and muscle transcriptome (**Figures 4A, B**). To further investigate the involvement of these genes in the pathways, we selected genes/proteins involved in more than two pathways. Multiple interleukins (mainly *IL-1β* and *IL-6*, *IL-8 [CXCL-8]*) and chemokines (mainly *CCL-3*, *CXCL-2*, and *CCL-18*) were identified as being upregulated in the *V. mimicus* group (**Figures 4C–E**), indicating a cytokine-dominated activation that is indicative of a cytokine storm. We also observed up-regulation of *matrix metallopeptidase* (*MMP*) -9 and *MMP-13* in all three bioinformatics analyses (**Figures 4C–E**). Notably, a protein network analysis of differentially expressed genes revealed that changes in inflammatory cytokines and collagen were centrally regulated by *MMP-9* and *MMP-13* (**Figure 4F**), suggesting a crucial role for Matrix metalloproteinases in the pathogenesis of *V. mimicus*-causing yellow catfish skin and muscle.

### 3.5 IL-17C and Nox1/2 may be the upstream of inflammation and tissue remodeling

**Figure 5:**
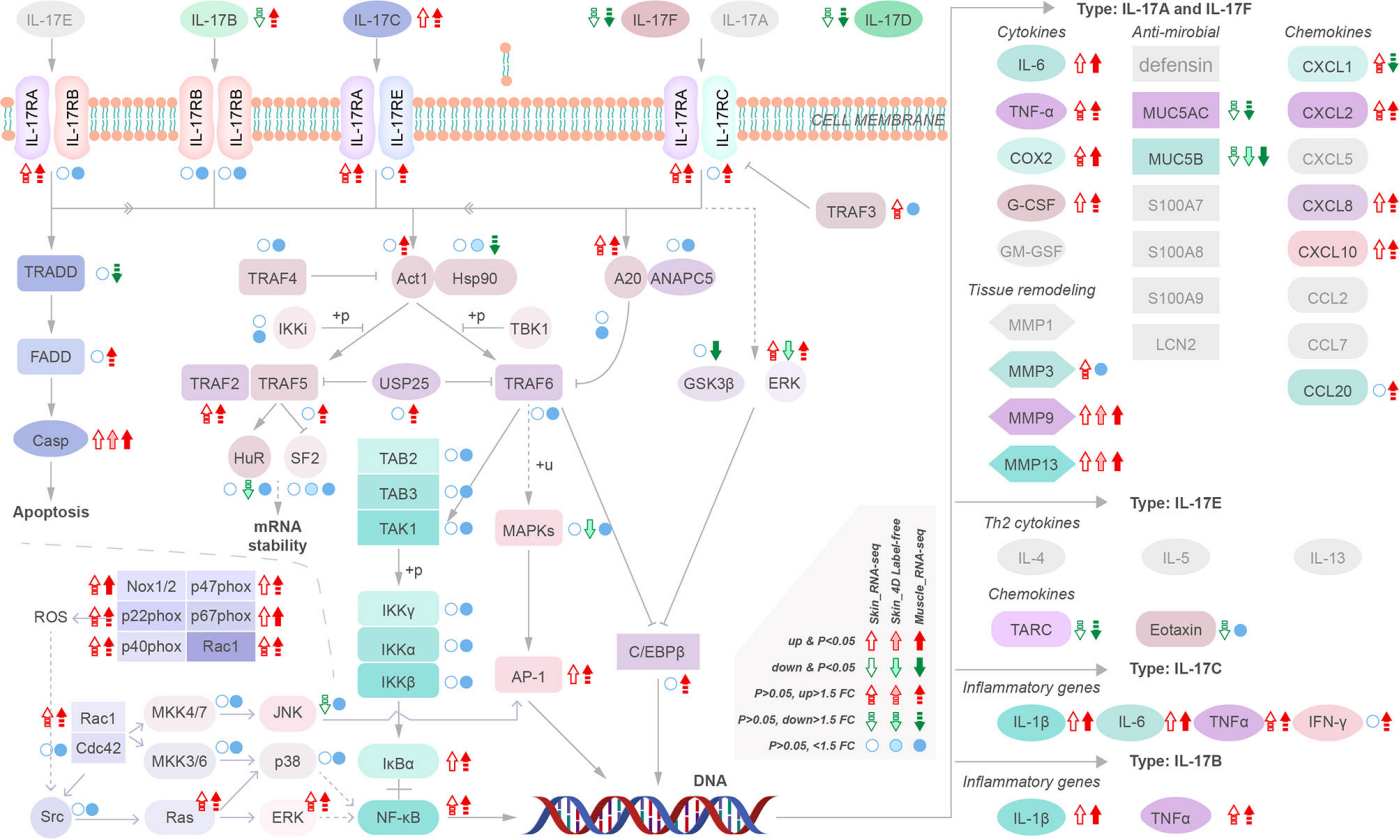
Transcriptional regulatory maps of genes upstream of the inflammatory storm and tissue remodeling.

We analyzed the regulatory pathways of MMP-9 & -13 and cytokines to identify their upstream regulatory targets in yellow catfish. Our results indicate that upregulation of *IL-17C* instead other *IL17s* may activate downstream cytokines and *MMP-9 & -13 via* activation of *NF-κB* (*NF-κB1, nuclear factor of kappa light polypeptide gene enhancer in B-cells 1*) and *AP-1* (*FOS like 1, AP-1 transcription factor subunit b*) and inhibition of antimicrobials such as *Mucin (MUC) 5AC* and *MUC 5B* (**Figure 5**). However, the intermediate regulatory process remains unclear. Moreover, the activation of *NADPH oxidase*, specifically Nox 1/2, is also observed, which can induce upregulation of *NF-κB1* and *FOSL-1* (**Figure 5**). These findings suggest that multiple pathways are involved in the upregulation of *MMP-9 & -13* and cytokines providing a potential target for intervention in the disease.

### 3.6 Confirmation of cytokine storm and tissue remodeling with qPCR and ELISA

**Figure 6:**
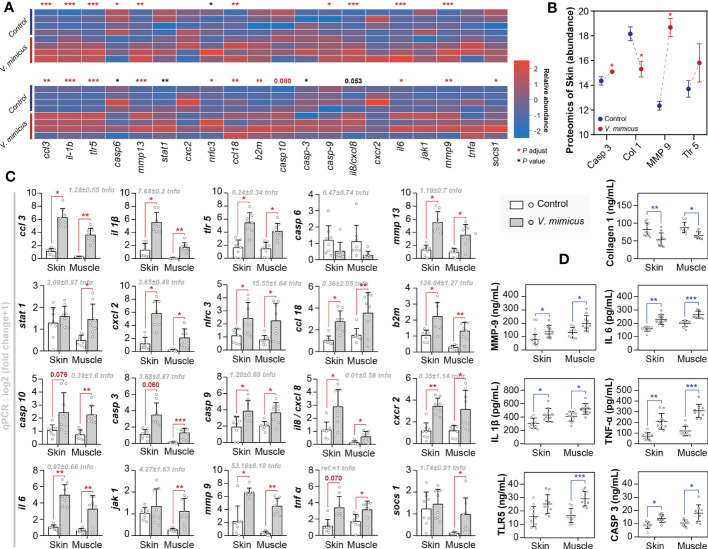
Gene expression in yellow catfish after *V. mimicus* infection. **(A)** Relative expression of qPCR genes corresponding to transcripts in skin and muscle (n=4); **(B)** Abundance of relevant annotated proteins in the skin proteome (n=3); **(C)** Results of qPCR assays for gene expression (n=8); **(D)** Results of ELISA assays for relevant proteins (n=10). ∗ P < 0.05, ∗∗ P < 0.01, *** P < 0.001 represents a significant or highly significant difference between groups.

We employed pathological analysis and integrated bioinformatic revealed the existence of cytokine storms and tissue remodeling, as evidenced by the upregulation of critical genes and proteins involved in these processes. Moreover, to validate our findings, we conducted qPCR (n=8) and ELISA (n=10) tests on a more extensive set of skin and muscle samples from yellow catfish infected with *V. mimicus*. The present study sought to validate the pathways of differential expression identified in our previous histological and bioinformatics investigations through the implementation of 20 gene-level tests and 7 protein-level tests (**Figure 6**). Specifically, we focused on the Toll-like receptor signaling pathway, IL-17 signaling pathway, cytokine-cytokine receptor interaction, cell death pathway, and collagen and enzyme-related pathways. The expression profiles of the annotated genes and proteins related to these pathways are presented in **Figures 6A, B**. The qPCR results were consistent with the transcriptomic results, as shown in **Figures 6A, B**. The ELISA results confirmed the label-free proteomics results and showed that the proteins in muscle had similar expression trends to those in the skin (**Figures 6B, D**). Our findings indicate that inflammation, necrosis, and tissue remodeling in the skin and muscle are associated with the regulatory pathways of these genes. Notably, the qPCR assay identified *β2 microglobulin* (*b2m*) (138.04 + 1.27-fold), *mmp-9* (53.16 ± 0.18-fold), *NLR family CARD domain-containing protein* (*nlrc*) *-3* (15.55 ± 1.64-fold), and *il-1β* (7.68 ± 0.2-fold) as highly expressed genes, highlighting their importance in the regulation of these pathways (**Figure 6C**).

## 4 Discussion

Outbreaks of disease caused by the pathogenic bacterium *V. mimicus* has resulted serious economic losses in catfish aquaculture. Despite its economic and ecological importance, the mechanism of *V. mimicus* infection is largely unknown in yellow catfish. To address this knowledge gap, we comprehensively analyzed integrated bioinformatics to reveal the mechanism of *V. mimicus* infection in yellow catfish. By histopathological analysis, we found that infected fish exhibited pathological changes dominated by necrosis and inflammation in their skin and muscles. Additionally, infected yellow catfish showed significant tissue remodeling, manifested by degeneration of the perimysium, and the lesion entered the muscle along the endomysium. Only the vacuolated structures of the endomysium were observed. Picrosirius red staining revealed that the collagen in the perimysium and the foci of the muscle bundles changed from type I to a mixture of type I and type III collagens. We further conducted eukaryotic transcriptomic and 4D Label-free analyses, which revealed a predominantly inflammatory pathway response, a cytokine storm in skin and muscle, and a downregulation of tight junctions and other pathways. We observed significant upregulation of IL-1β, IL-6, chemokines, MMP-9, and MMP-13, while significant downregulation of col1a and other genes. Our further analysis revealed that these pathways were differentially regulated with MMP-9 and MMP-13 as the potential core regulators of cytokine and tissue remodeling pathways. Moreover, MMP and cytokine-related genes may be held by the upregulation of *NF-κB1* and *FOSL-1* induced by IL-17C and NADPH oxidase. We validated the relevant regulatory pathways by qPCR and ELISA in expanded samples. This study provides new insights into the complex immune response to *V. mimicus* infection in yellow catfish.

The cytokine storm is a well-known phenomenon characterized by clinical manifestations from an overactivated immune system. Here, we report a significant increase of cytokines, mainly IL-1β, IL-6, and many chemokines, on the skin and muscles of yellow catfish during *V. mimicus* infection, suggesting the occurrence of a cytokine storm. This phenomenon involves a positive feedback loop between cytokine release and cell death pathways, where specific cytokines, PAMPs, and DAMPs activate inflammatory cell death, leading to further cytokine secretion (26). For instance, TNF-α and IFN-γ have been shown to cause a lethal cytokine shock in mice, which mirrors the tissue damage and inflammation (27). In our study, inflammation and necrosis were observed to coexist in the skin and muscle with a high expression of interleukins and chemokines. While we do not have direct evidence to suggest a closed loop between necrosis and cytokines, we interpret this phenomenon as a cytokine storm. Another feature of the cytokine storm is a disturbance in its regulatory mechanisms, which is also observed in our study. Normally, inhibitory cytokines regulate excessive immune responses. Our research findings indicate the presence of negative regulators, namely the suppressor of cytokine signaling (SOCS)-1 protein, which effectively inhibits cytokine signaling through a negative feedback loop (28). However, our observations also found that the SOCS-1 protein might be inadequate in suppressing its target Jak-STAT pathway (29), suggesting that the negative feedback regulation in yellow catfish is inadequate and resulting in an imbalance in the immune network (28). Additionally, *V. mimicus* sensitization of yellow catfish often results in square-shaped hemorrhagic ulcers on the body surface and causes more than 90% mortality in 3-5 days, consistent with aseptic shock. This shock is a subset of sepsis characterized by hypotension and significantly increased mortality risk (30). The cytokine storm observed in our study further indicates that *V. mimicus* can elicit a severe immune response in the host, resulting in tissue damage and a high mortality rate. Therefore, understanding the mechanisms of cytokine storm caused by *V. mimicus* infection could provide insights into developing effective treatments against this pathogen.

This study revealed that *V. mimicus* infection leads to perimysium extension into inflammatory necrosis and the formation of a collagen remodeling network. Tissue remodeling is an essential process responsible for developing and maintaining tissues and organs (31). A previous study has demonstrated that myocardial infarction induces tissue remodeling in a three-phase process: 1) massive necrosis in the infarcted area, 2) inflammatory cells replaced by myofibroblasts that produce large amounts of collagen, and 3) myofibroblast numbers reduced by apoptosis, allowing for infiltration by endothelial cells and cardiomyocytes (32). In this study, the myofibrillar lesions were encapsulated by collagen, indicating the second step of tissue remodeling. However, in most cases, tissue remodeling is pathological, resulting in a large amount of fibrous tissue (33). We found that collagen in the perimysium and the foci of the muscle bundles shifted from the tougher type I collagen to the sparser type III collagen (34), and bioinformatics analysis revealed a significant decrease in type I collagen. We hypothesize that *V. mimicus* may have utilized this process to assist its growth. *Vibrio mimicus* was found to prefer a purine and short peptide-rich microenvironment for growth in our study (data not shown), thus suggesting that type I collagen is broken down into short peptides, some of which are used to resynthesize type III collagen. At the same time, the rest is utilized by *V. mimicus*. In addition, *V. mimicus* contains collagenase for degrading collagen to form short peptides (35), and the collagenase of the host found in this study may also be involved in this process. The core genes revealed in this study, MMP-9, and MMP-13, play a crucial role in the degradation of extracellular matrix components that allow cellular movement, migration, and rearrangement (36). Moreover, it should be noted that MMP-9 and MMP-13 play a pivotal role not only in regulating the degradation of collagen but also in displaying degradative impacts on diverse non-collagenous extracellular matrix substrates, including aggrecan, elastin, fibronectin, and laminin (37). The tissue remodeling process possesses the potential to enhance the infectivity of *V. mimicus* by effectively promoting the infiltration of the bacteria between tissues through the reduction of intercellular connections. As a result, this tissue remodeling mechanism may furnish a favorable environment for *V. mimicus* to thrive and enhance its pathogenicity.

MMPs are a family of over 25 zinc-dependent enzymes that are capable of cleaving numerous structural components. Their substrates include not only extracellular matrix proteins, but also ligand and receptor substrates such as cytokines, chemokines, growth factors, and adhesion molecules (38). Based on their collagen-degrading properties, MMP-9 belongs to the gelatinase B subgroup, while MMP-13 belongs to the collagenase 3 subgroup. Several studies have demonstrated the significant regulatory roles of MMP-9 and -13 on non-matrix extracellular proteins (39). These two enzymes have been implicated in various inflammatory diseases, infections, and cancers (40). Specifically, MMP-9 has been shown to target CXCL5, IL-1β, IL2-R, plasminogen, pro-TNF-α, SDF-1, and TGF-β (36), while MMP-13’s targets include Casein, plasminogen activator 2, proMMP-9 and -13, and SDF-1 (37). In addition, Ke et al. conducted a detailed genetic evolution and structural analysis of MMP9 in yellow catfish and revealed its significant role in innate immunity against *Aeromonas hydrophila* infection (41), suggesting the conservation of MMP’s function in yellow catfish. Therefore, the discovery of MMP-9 and MMP-13 in this study suggests their potential core regulatory roles in cellular necrosis, inflammatory response, and tissue remodeling after *V. mimicus* infection (42–44). Moreover, Luchian et al. indicated that MMP-13 also participates in certain anti-inflammatory activities intracellularly (39). However, given our discussion on the inflammatory storm, the overall inflammatory response in the skin and muscles of yellow catfish presents a dysregulated and amplified state, which may be an important reason for the rapid death of yellow catfish caused by *V. mimicus* infection.

Therefore, based on the present study’s findings, which identified the two central phenomena of the inflammatory storm and tissue remodeling, and a comprehensive analysis of gene networks, MMP-9 and MMP-13 were identified as core genes that play critical roles in regulating essential biological processes. Moreover, the study suggests multiple pathways may control their upstream regulation, such as *NF-κB1* and *FOSL-1* activation due to IL-17C and Nox-1/2-based NADPH oxidase (45, 46). Previous study has suggested that skin inflammation in mammals, including mice, is regulated by transcription factors such as *AP-1* (*Fos/Jun*), *NF-κB*, *NFATs*, and *STATs* (47). Specifically, NF-κB translocates into the nucleus and induces the expression of genes related to a wide range of biological functions, such as cytokines, oxidant-free radicals, ultraviolet irradiation, and bacterial or viral products (48). On the other hand, FOSL-1 is involved in DNA-binding transcription factor activity and RNA polymerase II transcription regulatory region sequence-specific DNA binding (47). Our research has found that the regulation of skin and muscle inflammation in the yellow catfish is similar to that in mammals, with an upregulation of *NF-κB1*, *FOSL-1*, and even *STATs* during the inflammatory response. Therefore, upstream regulation of MMPs, interleukins, and chemokines may be associated with these proteins, but whether the pathways regulating these downstream in yellow catfish are conserved remains unknown. Additionally, Further investigation of their mechanisms could provide insights leading to new treatments for various pathological conditions. Several drugs, including Doxycycline, Minocycline, and TISAM, have shown good inhibition of MMP-9 or MMP-13 (49). Of note, Doxycycline has demonstrated a good killing effect on some *V. mimicus* strains (50), and the research on the therapeutic development and formulation of *V. mimicus* is worth conducting.

## Data availability statement

The datasets supporting the conclusions of this article are included in the report. The data used for transcriptome analysis could obtained in the Genebank (NCBI: CKSK01[SRR23641111], CKSK02 [SRR23641111], CKSK03 [SRR23641111], CKSK04 [SRR23698941], VMSK01 [SRR23698942], VMSK02 [SRR23698942], VMSK03 [SRR23700451], VMSK04 [SRR23701013], CKMU01 [SRR23701949], CKMU02 [SRR23701949], CKMU03 [SRR23711623], CKMU04 [SRR23711624], VMMU01 [SRR23724919], VMMU02 [SRR23725279], VMMU03 [SRR23725280], and VMMU04 [SRR23725281]).

## Ethics statement

All animal handling procedures were approved by the Animal Care and Use Committee of Sichuan Agricultural University and followed the guidelines for animal experiments of Sichuan Agricultural University under permit number 2020103001.

## Author contributions

YF: writing, methodology and investigation. JW: investigation and formal analysis. WF: methodology and investigation. YG: writing - review and editing, resources, funding acquisition and supervision. XH: methodology and investigation. PO: conceptualization, methodology, writing - review and editing. DC: formal analysis, conceptualization and methodology. HG: writing - review and editing. HD: data curation and formal analysis. WL: methodology and formal analysis. ZCZ: data curation and resources. All authors contributed to the article and approved the submitted version.

## Glossary

**Table:**

*ACKR2*	*atypical chemokine receptor 2*
*acp5a*	*acid phosphatase 5a, tartrate resistant, transcript variant X1*
*act-3*	*adenylate cyclase type 3-like, transcript variant X1*
*ACTN1*	*alpha-actinin-1 isoform X2*
*ACTN4*	*alpha-actinin-4 isoform X1*
*agt*	*angiotensinogen*
*b2ml*	*beta-2-microglobulin, like*
*bada*	*BCL2 associated agonist of cell death a, transcript variant X1*
*CAMK2*	*calcium/calmodulin-dependent protein kinase type II subunit beta isoform X1*
*camk2d2*	*calcium_calmodulin-dependent protein kinase (CaM kinase) II delta 2, transcript variant X1*
*CAPN2L*	*calpain-2 catalytic subunit-like*
*card9*	*caspase recruitment domain family, member 9, transcript variant X1*
*CAS3B*	*caspase-3b*
*casp23*	*caspase 23, apoptosis-related cysteine peptidase, transcript variant X1*
*Casp8*	*caspase-8-like, transcript variant X1*
*ccl18*	*C-C motif chemokine 18-like*
*ccl18x2*	*C-C motif chemokine 18-like, transcript variant X2*
*ccl19a.1*	*chemokine (C-C motif) ligand 19a, tandem duplicate 1*
*ccl3*	*C-C motif chemokine 3-like*
*CDC42*	*cell division control protein 42 homolog isoform X2*
*COL1A*	*collagen alpha-1(IV) chain*
*col1a1a*	*collagen, type I, alpha 1a*
*col1a2*	*collagen, type I, alpha 2, transcript variant X1*
*col3l*	*collagenase 3-like*
*CRKL*	*crk-like protein*
*CTNNA1X2*	*catenin beta-1 isoform X2*
*cxc5/6*	*alveolar macrophage chemotactic factor-like*
*cxcl8a*	*chemokine (C-X-C motif) ligand 8a*
*cxcl8b.3*	*chemokine (C-X-C motif) ligand 8b, duplicate 3, transcript variant X1*
*CXCR1*	*C-X-C chemokine receptor type 1*
*cxcr2*	*chemokine (C-X-C motif) receptor 2, transcript variant X2*
*cytib245*	*cytochrome b-245 heavy chain*
*ddx58*	*DEAD (Asp-Glu-Ala-Asp) box polypeptide 58, transcript variant X1*
*dhx58*	*DEXH (Asp-Glu-X-His) box polypeptide 58, transcript variant X2*
*epsilon-l*	*14-3-3 protein epsilon-like*
*f3a*	*coagulation factor IIIa*
*fgfr3*	*fibroblast growth factor receptor 3, transcript variant X2*
*fosl1b*	*FOS like 1, AP-1 transcription factor subunit b*
*gbp2*	*guanylate binding protein 2, transcript variant X1*
*GNAIA*	*guanine nucleotide-binding protein G(i) subunit alpha*
*GNAIA1*	*guanine nucleotide-binding protein G(i) subunit alpha-1*
*GNAIA11L*	*guanine nucleotide-binding protein subunit alpha-11-like*
*GNAIA1B*	*guanine nucleotide binding protein (G protein), beta polypeptide 1b*
*GRB2B*	*growth factor receptor-bound protein 2b*
*gsk3bb*	*glycogen synthase kinase 3 beta, genome duplicate b, transcript variant X1*
*HSP70B4*	*heat shock 70 kDa protein 4b*
*HSP90A1*	*heat shock protein HSP 90-alpha 1*
*hsp90aa1.2*	*heat shock protein 90, alpha (cytosolic), class A member 1, tandem duplicate 2*
*ifnphi3*	*interferon phi 3*
*il1b*	*interleukin 1, beta*
*il1bl*	*interleukin-1 beta-like*
*IL1R2*	*interleukin-1 receptor type 2*
*il1βl*	*interleukin-1 beta-like*
*il1βlt2*	*interleukin-1 beta-like, transcript variant X2*
*il6*	*interleukin 6 (interferon, beta 2)*
*irf9*	*interferon regulatory factor 9, transcript variant X2*
*irg1l*	*immunoresponsive gene 1, like*
*LBP-like*	*lipopolysaccharide-binding protein-like*
*LOC113635103*	*uncharacterized LOC113635103*
*LOC125145525*	*uncharacterized LOC125145525*
*map3k5*	*mitogen-activated protein kinase kinase kinase 5, transcript variant X1*
*MAPK3*	*mitogen-activated protein kinase 3 isoform X2*
*MAPK6*	*dual specificity mitogen-activated protein kinase kinase 6 isoform X4*
*mmp13*	*collagenase*
*mmp13b*	*matrix metallopeptidase 13b*
*MMP13L*	*collagenase 3-like*
*mmp13x2*	*collagenase 3-like, transcript variant X2*
*mmp9*	*matrix metallopeptidase 9*
*mmp9l*	*matrix metalloproteinase-9-like*
*mx1l*	*interferon-induced GTP-binding protein Mx1-like*
*mx2l*	*interferon-induced GTP-binding protein Mx2-like*
*mx2lx1*	*interferon-induced GTP-binding protein Mx2-like, transcript variant X1*
*mx3l*	*interferon-induced GTP-binding protein Mx3-like*
*mxe*	*myxovirus (influenza virus) resistance E, transcript variant X1*
*ncf1*	*neutrophil cytosolic factor 1, transcript variant X1*
*ncf2*	*neutrophil cytosolic factor 2*
*nfkbiab*	*nuclear factor of kappa light polypeptide gene enhancer in B-cells inhibitor, alpha b*
*nlrp3l*	*NLR family CARD domain-containing protein 3-like*
*nox1*	*NADPH oxidase 1*
*NRAS*	*GTPase NRas*
*pif2l*	*permeability factor 2-like*
*PIP3B2*	*1-phosphatidylinositol 4,5-bisphosphate phosphodiesterase beta-3 isoform X2*
*PPP2R*	*serine/threonine-protein phosphatase 2A catalytic subunit beta isoform*
*PPP2RB*	*serine/threonine-protein phosphatase 2A 55 kDa regulatory subunit B alpha isoform isoform X1*
*PRKCB*	*protein kinase C, beta a isoform X2*
*ptkl*	*tyrosine-protein kinase Lyn-like, transcript variant X1*
*ptx3a*	*pentraxin 3, long a, transcript variant X1*
*raf1b*	*Raf-1 proto-oncogene, serine_threonine kinase b*
*RASC3TS1B*	*ras-related C3 botulinum toxin substrate 1b*
*RHOAD*	*rho-related GTP-binding protein RhoA-D*
*RRAS1B*	*ras-related protein Rap-1b*
*socs1a*	*suppressor of cytokine signaling 1a*
*socs1b*	*suppressor of cytokine signaling 1b, transcript variant X2*
*socs3a*	*suppressor of cytokine signaling 3a*
*socs3b*	*suppressor of cytokine signaling 3b*
*SRCLX3*	*proto-oncogene tyrosine-protein kinase Src-like isoform X3*
*stat2*	*signal transducer and activator of transcription 2, transcript variant X1*
*STAT3*	*signal transducer and activator of transcription 3 isoform X2*
*tap2-like*	*antigen peptide transporter 2-like*
*tapasin*	*tapasin*
*tgfb3*	*transforming growth factor, beta 3, transcript variant X1*
*THBS1*	*thrombospondin-1*
*TLN1X2*	*talin-1 isoform X2*
*tln2b*	*talin 2b, transcript variant X1*
*tnfb*	*tumor necrosis factor b (TNF superfamily, member 2)*
*tnfsf13b*	*TNF superfamily member 13b, transcript variant X2*
*traf3*	*TNF receptor-associated factor 3, transcript variant X2*
*vegfab*	*vascular endothelial growth factor Ab, transcript variant X1*
*vegfd*	*vascular endothelial growth factor D, transcript variant X1*
*ywhaqa*	*tyrosine 3-monooxygenase_tryptophan 5-monooxygenase activation protein, theta polypeptide a*
*ZFA89*	*claudin-like protein ZF-A89 isoform X1*

## References

[1] Amaro C Fouz B Sanjuán E Romalde JL. Vibriosis. In: Climate change and infectious fish diseases. Wallingford UK: CABI (2020). p. 182–210.

[2] Skandalos I Christou K Psilas A Moskophidis M Karamoschos K. Mycotic abdominal aortic aneurysm infected by vibrio mimicus: report of a case. Surg Today (2009) 39(2):141–3. doi: 10.1007/s00595-008-3808-5 19198993

[3] Elgendy MY Abdelsalam M Kenawy AM Ali SE. Vibriosis outbreaks in farmed Nile tilapia (Oreochromis niloticus) caused by vibrio mimicus and V. Cholerae. Aquaculture Int (2022) 30(5):2661–77. doi: 10.1007/s10499-022-00921-8

[4] Gao H Hou L Li J Huang A. The green fluorescent protein markers of vibrio mimicus and their dynamic distribution in infected grass carps. J Fish Chin (2015) 39:557–65. doi: 10.11964/jfc.20141209594

[5] Geng Y Liu D Han S Zhou Y Wang KY Huang XL. Outbreaks of vibriosis associated with *Vibrio mimicus* in freshwater catfish in China. Aquaculture (2014) 433:82–4. doi: 10.1016/j.aquaculture.2014.05.053

[6] Yosra M Mahmoud M Ebtsam S Sabah I Ahmad. Subclinical vibriosis in red swamp crayfish, *Procambarus clarkii* . Int J Fish Aquat Stud (2016) 4(3):119–23.

[7] Jin-yu S Wen-lin Y Dong Q Wen L Zheng C Zhi-hua S. Studies on the pathogens of bacterial diseases of eriocheir sinensis. J Fishery Sci China (2000) 7(3):89–92.

[8] Fu Y Zhang Y-A Shen J Tu J. Immunogenicity study of ompu subunit vaccine against vibrio mimicus in yellow catfish, pelteobagrus fulvidraco. Fish Shellfish Immunol (2021) 108:80–5. doi: 10.1016/j.fsi.2020.11.030 33285164

[9] Bureau of Fisheries MoAaRA. China Fishery statistics yearbook 2022. Beijing: China Agriculture Press (2022).

[10] Mohamad N Amal MNA Yasin ISM Saad MZ Nasruddin NS Al-saari N. Vibriosis in cultured marine fishes: a review. Aquaculture (2019) 512:734289. doi: 10.1016/j.aquaculture.2019.734289

[11] Miyoshi S-i Toko N Dodo T Nanko A Mizuno T. Second extracellular protease mediating maturation of vibrio mimicus hemolysin. World J Microbiol Biotechnol (2022) 38(12):241. doi: 10.1007/s11274-022-03436-9 36271946

[12] Ganie HA Choudhary A Baranwal S. Structure, regulation, and host interaction of outer membrane protein U (Ompu) of vibrio species. Microb Pathog (2022) 162:105267. doi: 10.1016/j.micpath.2021.105267 34718127

[13] Zhao R. Functional analysis of sialic acid cetabolism system of vibrio mimicus that isolated from catfish. [master’s thesis]. Chengdu: Sichuan Agric Univ (2020). doi: 10.27345/d.cnki.gsnyu.2020.000119

[14] Yu Z Geng Y Wang K Chen D Huang X Ou Y. Complete genome sequence of *Vibrio mimicus* strain Sccf01 with potential application in fish vaccine development. Virulence (2017) 8(6):1028–30. doi: 10.1080/21505594.2016.1250996 PMC5626196 27763808

[15] Yu Z Wang E Geng Y Wang K Chen D Huang X. Multiplex genome editing by natural transformation in *Vibrio mimicus* with potential application in attenuated vaccine development. Fish Shellfish Immunol (2019) 92:377–83. doi: 10.1016/j.fsi.2019.06.025 31202969

[16] Xiang F Tian Z Feng Y Qin Z Peng K Ouyang P. Construction and biological characterization of vibrio mimicus’s metalloprotease gene prtv-deletion strain. Microbiol China (in Chinese) (2023), 1–15. doi: 10.13344/j.microbiol.china.220968

[17] Lee J-H Ahn S-H Kim S-H Choi Y-H Park K-J Kong I-S. Characterization of vibrio mimicus phospholipase a (Phla) and cytotoxicity on fish cell. Biochem Biophys Res Commun (2002) 298(2):269–76. doi: 10.1016/S0006-291X(02)02434-8 12387827

[18] Tao L Hu D Zhang J Tao H Lu J. Differential proteome analysis of spleen and intestinal mucosa tissue from carassius auratus infected by vibrio mimicus. J Fisheries China (2018) 42(4):596–604. doi: 10.11964/jfc.20160810510

[19] Li J-N Zhao Y-T Cao S-L Wang H Zhang J-J. Integrated transcriptomic and proteomic analyses of grass carp intestines after vaccination with a double-targeted DNA vaccine of vibrio mimicus. Fish Shellfish Immunol (2020) 98:641–52. doi: 10.1016/j.fsi.2019.10.045 31678536

[20] Chen C Geng Y Wang K Yu Z Chen D Ouyang P. Dynamic distribution of vibrio mimicus in infected yellow Catfish(Pelteobagrus fulvidraco) and its hispathological changes. South China Fisheries Sci (in Chinese) (2017) 13(1):10–8. doi: 10.3969/j.issn.2095-0780.2017.01.002

[21] Yu Z Wang E Geng Y Wang K Chen D Huang X. Complete genome analysis of *Vibrio mimicus* strain Sccf01, a highly virulent isolate from the freshwater catfish. Virulence (2020) 11(1):23–31. doi: 10.1080/21505594.2019.1702797 31826705 PMC6961728

[22] Baums C Hermeyer K Leimbach S Adamek M Czerny C-P Hörstgen-Schwark G. Establishment of a model of streptococcus iniae meningoencephalitis in Nile tilapia (Oreochromis niloticus). J Comp Pathol (2013) 149(1):94–102. doi: 10.1016/j.jcpa.2012.10.003 23218409

[23] Grabherr M Haas B Yassour M Levin J Thompson D Amit I. Full-length transcriptome assembly from rna-seq data without a reference genome. Nat Biotechnol (2011) 29:644–52. doi: 10.1038/nbt.1883 PMC3571712 21572440

[24] Sun M Jiang K Zhang F Zhang D Shen A Jiang M. Effects of various salinities on na+-K+-Atpase, Hsp70 and Hsp90 expression profiles in juvenile mitten crabs, eriocheir sinensis. Gen Mol Res (2012) 11(2):978–86. doi: 10.4238/2012.April.19.3 22576924

[25] Livak KJ Schmittgen TD. Analysis of relative gene expression data using real-time quantitative pcr and the 2(-delta delta C(T)) method. Methods (2001) 25(4):402–8. doi: 10.1006/meth.2001.1262 11846609

[26] Karki R Kanneganti T-D. The ‘Cytokine storm’: molecular mechanisms and therapeutic prospects. Trends Immunol (2021) 42(8):681–705. doi: 10.1016/j.it.2021.06.001 34217595 PMC9310545

[27] Karki R Sharma BR Tuladhar S Williams EP Zalduondo L Samir P. Synergism of tnf-A and ifn-Γ triggers inflammatory cell death, tissue damage, and mortality in sars-Cov-2 infection and cytokine shock syndromes. Cell (2021) 184(1):149–68.e17. doi: 10.1016/j.cell.2020.11.025 33278357 PMC7674074

[28] Tisoncik JR Korth MJ Simmons CP Farrar J Martin TR Katze MG. Into the eye of the cytokine storm. Microbiol Mol Biol Rev (2012) 76(1):16–32. doi: 10.1128/MMBR.05015-11 22390970 PMC3294426

[29] Liau NPD Laktyushin A Lucet IS Murphy JM Yao S Whitlock E. The molecular basis of Jak/Stat inhibition by Socs1. Nat Commun (2018) 9(1):1558. doi: 10.1038/s41467-018-04013-1 29674694 PMC5908791

[30] Hotchkiss RS Moldawer LL Opal SM Reinhart K Turnbull IR Vincent J-L. Sepsis and septic shock. Nat Rev Dis Primers (2016) 2(1):1–21. doi: 10.1038/nrdp.2016.45 PMC5538252 28117397

[31] Pinet K McLaughlin KA. Mechanisms of physiological tissue remodeling in animals: manipulating tissue, organ, and organism morphology. Dev Biol (2019) 451(2):134–45. doi: 10.1016/j.ydbio.2019.04.001 30974103

[32] Shinde AV Frangogiannis NG. Fibroblasts in myocardial infarction: a role in inflammation and repair. J Mol Cell Cardiol (2014) 70:74–82. doi: 10.1016/j.yjmcc.2013.11.015 24321195 PMC3995820

[33] Talman V Ruskoaho H. Cardiac fibrosis in myocardial infarction–from repair and remodeling to regeneration. Cell Tissue Res (2016) 365:563–81. doi: 10.1007/s00441-016-2431-9 PMC5010608 27324127

[34] Zhao J Hu L Gong N Tang Q Du L Chen L. The effects of macrophage-stimulating protein on the migration, proliferation, and collagen synthesis of skin fibroblasts in vitro and in vivo. Tissue Eng Part A (2015) 21(5-6):982–91. doi: 10.1089/ten.tea.2013.0726 25315688

[35] Wang Y Su H-N Cao H-Y Liu S-M Liu S-C Zhang X. Mechanistic insight into the fragmentation of type I collagen fibers into peptides and amino acids by a vibrio collagenase. Appl Environ Microbiol (2022) 88(7):e01677–21. doi: 10.1128/aem.01677-21 PMC9004396 35285716

[36] McCawley LJ Matrisian LM. Matrix metalloproteinases: they're not just for matrix anymore. Curr Opin Cell Biol (2001) 13(5):534–40. doi: 10.1016/S0955-0674(00)00248-9 11544020

[37] Cui N Hu M Khalil RA. Biochemical and biological attributes of matrix metalloproteinases. Prog Mol Biol Transl Sci (2017) 147:1–73. doi: 10.1016/bs.pmbts.2017.02.005 28413025 PMC5430303

[38] Franco C Patricia H-R Timo S Claudia B Marcela H. Matrix metalloproteinases as regulators of periodontal inflammation. Int J Mol Sci (2017) 18(2):440. doi: 10.3390/ijms18020440 28218665 PMC5343974

[39] Luchian I Goriuc A Sandu D Covasa M. The role of matrix metalloproteinases (Mmp-8, mmp-9, mmp-13) in periodontal and peri-implant pathological processes. Int J Mol Sci (2022) 23(3):1806. doi: 10.3390/ijms23031806 35163727 PMC8837018

[40] Segura-Valdez L Pardo A Gaxiola M Uhal BD Becerril C Selman M. Upregulation of gelatinases a and b, collagenases 1 and 2, and increased parenchymal cell death in copd. Chest (2000) 117(3):684–94. doi: 10.1378/chest.117.3.684 10712992

[41] Ke F Wang Y Hong J Xu C Chen H Zhou S-B. Characterization of mmp-9 gene from a normalized cdna library of kidney tissue of yellow catfish (Pelteobagrus fulvidraco). Fish Shellfish Immunol (2015) 45(2):260–7. doi: 10.1016/j.fsi.2015.04.012 25910849

[42] Opdenakker G Van den Steen PE Dubois B Nelissen I Van Coillie E Masure S. Gelatinase b functions as regulator and effector in leukocyte biology. J leukocyte Biol (2001) 69(6):851–9. doi: 10.1189/jlb.69.6.851 11404367

[43] Nguyen DP Li J Tewari AK. Inflammation and prostate cancer: the role of interleukin 6 (Il-6). BJU Int (2014) 113(6):986–92. doi: 10.1111/bju.12452 24053309

[44] Turner RJ Sharp FR. Implications of Mmp9 for blood brain barrier disruption and hemorrhagic transformation following ischemic stroke. Front Cell Neurosci (2016) 10:56. doi: 10.3389/fncel.2016.00056 26973468 PMC4777722

[45] Zenobia C Hajishengallis G. Basic biology and role of interleukin-17 in immunity and inflammation. Periodontol 2000 (2015) 69(1):142–59. doi: 10.1111/prd.12083 PMC4530463 26252407

[46] Roy K Wu Y Meitzler JL Juhasz A Liu H Jiang G. Nadph oxidases and cancer. Clin Sci (2015) 128(12):863–75. doi: 10.1042/CS20140542 25818486

[47] Uluckan O Guinea-Viniegra J Jimenez M Wagner EF. Signalling in inflammatory skin disease by ap-1 (Fos/Jun). Clin Exp Rheumatol (2015) 33(4 Suppl 92):S44–S9. 26458100

[48] Ghosh S Hayden M. Celebrating 25 years of nf-Kb research. Immunol Rev (2012) 246(1):5. doi: 10.1111/j.1600-065X.2012.01111.x 22435544 PMC3313446

[49] Hu J Van den Steen PE Sang Q-XA Opdenakker G. Matrix metalloproteinase inhibitors as therapy for inflammatory and vascular diseases. Nat Rev Drug Discovery (2007) 6(6):480–98. doi: 10.1038/nrd2308 17541420

[50] Lin L Feng D Pan X Yao J Yin W Cao Z. Identification, virulence-related factors, and antimicrobial susceptibility of vibrio mimicus from yellow catfish, pelteobagrus fulvidrac. Acta Hydrobiologica Sin (in Chinese) (2020) 4):799–810. doi: 10.7541/2020.096

