# Supplementary material for: Integrated bioinformatics identifies key mediators in cytokine storm and tissue remodeling during Vibrio mimicus infection in yellow catfish (Pelteobagrus fulvidraco)
Source: Front Immunol. 2023 May 22;14:1172849. doi: 10.3389/fimmu.2023.1172849 (PMC10239856; doi:10.3389/fimmu.2023.1172849)
Supplement: Supplementary file 1 [file DataSheet_1.docx]

**Supplementary Material**

**Integrated bioinformatics identifies key mediators in cytokine storm and tissue remodeling during *Vibrio mimicus* infection in yellow catfish *(Pelteobagrus fulvidraco)***

Yang Feng^1†^, Jiao Wang^1†^, Wei Fan^1†^, Yi Geng^1*^, Xiaoli Huang^2^, Ping Ouyang^1^, Defang Chen^2^, Hongrui Guo^1^, Huidan Deng^1^, Weimin Lai^1^, Zhicai Zuo^1^

1 College of Veterinary Medicine, Sichuan Agricultural University, Wenjiang 611130, Sichuan, China.

2 Department of Aquaculture, College of Animal Science & Technology, Sichuan Agricultural University, Wenjiang 611130, Sichuan, China.

* CORRESPONDENCE: Yi Geng. E-mail: gengyisicau@126.com

^†^ These authors have contributed equally to this work

**Supplementary Table 1.** Primer sequences for qPCR.

| Genes | Forward (5'-3') | Reverse (5'-3') | Length（bp） | Ta (℃) | Ref./NCBI ID |
| --- | --- | --- | --- | --- | --- |
| *il-1β* | TAGGCATAGAGGAGGTAA | AAGGTGTTCAGGGAGTCA | 112 | 56 | (Wang et al. 2020) |
| *il-6* | CACTATCTTGCCCTGTTCCTG | TCGTGTTCTGTGTTCCTCCG | 185 | 62 | (Zhou et al. 2021) |
| *il-8* | CAAGCCAGCAATGACCTCT | CACTGAAGACAACCCAAGACT | 227 | 57 | (Zhang et al. 2020) |
| *nlrc-3* | GTGCCTGTGCTGCTGATAAACC | ACTCCTCCTTCTGTGGGTCATT | 157 | 61 | XM_047817140 |
| *tlr-5* | CTACCGAGCAAAGCAAGA | TGTAACTGGTGGAGATGG | 272 | 55 | (Zhong and Gao 2022) |
| *cxcr-2* | GGCTGCTGTTCTGTCCATTCC | CCAAGGATGTGACGAACGACTC | 147 | 61 | XM_047815440.1 |
| *cxc-2* | ATCTGCTGACCATTGCCCTT | GTCCTGACCTTCATCACCTT | 153 | 62 | (Zhou et al. 2021) |
| *ccl18* | TCTCTTCAACATCTTCATCAGCAGTCA | CAGGGATTGGTCGTGTCAGAAAGC | 150 | 61 | XM_047818166.1 |
| *ccl3* | TTCACATCCAGAGCAGCAGCATCA | TGAAGGATTGAAGGCAGGCGAGAA | 100 | 60 | XM_027134260.2 |
| *stat-1* | GTTTTGCAATGGAGAAGGACTT | CGCTCTGGGCGTTATGTG | 158 | 62 | (Zhou et al. 2021) |
| *jak-1* | GCACGCTGGTGATTCCTGACTG | CTCGCACTATGGCTCTGAAGAACG | 102 | 60 | (Cheng et al. 2020) |
| *socs-1* | AAACCGCACGCACTTCCGC | ACGACTCCTTGCTTCCCATAA | 255 | 62 | (Zhou et al. 2021) |
| *b2m* | GCTGATCTGCCATGTGAGTG | TGTCTGACACTGCAGCTGTA | 186 | 60 | (Li et al. 2019) |
| *mmp-9* | GGTGAGCTGGACCAACCAACAAT | GATCCCACTTCAGGTCTCCGTCA | 103 | 55 | (Ke et al. 2015) |
| *mmp-13* | TGTGGTGTGCCTGAAGTA | GTTACATTGCTCCAGACCTT | 161 | 61 | XM_027150769.2 |
| *casp-3* | TGACTTGCTGTGGTCCTCTT | AACGGAACTGATGTTGATGC | 141 | 61 | (Zhang et al. 2020) |
| *casp-6* | ATGAGGATGATAATGAGGTGGAGGAA | GAATAGAATCCCATTGCTGAAGAGT | 100 | 61 | XM_047812939.1 |
| *casp-9* | TGGTAGGCGAGACAGACACAGG | CAGACAAGCTGAGCGAGTCACTG | 126 | 61 | (Zhang et al. 2020) |
| *casp-10* | GGATGACCTGAGCACCCAAGAT | TGCCATCCAGCCTCTTCATCAG | 140 | 61 | XM_027164233.2 |
| *tnf-α* | AACCGAAAGGAAGCACAGAA | TCACGGCAATCGTTTAGGAG | 221 | 57 | (Zhang et al. 2020) |
| *b-actin* | TTCGCTGGAGATGATGCT | CGTGCTCAATGGGGTACT | 136 | 61 | (Zhang et al. 2020) |
| *18s rrna* | AGCTCGTAGTTGGATCTCGG | CGGGTATTCAGGCGAGTTTG | 196 | 60 | (Li et al. 2019) |
| *gapdh* | TTTCAGCGAGAGAGACCCAG | ATGACTCTCTTGGCACCTCC | 132 | 60 | (Li et al. 2019) |

**Supplementary Table 2.** Sample inspection and quality control

| **Organ** | **Group** | **Sample** | **Con (ng/μl)** | **Total RNA (μg)** | **OD260/280** | **OD260/230** | **RQN** | **Raw reads** | **Raw bases** | **Clean reads** | **Clean bases** | **Error rate (%)** | **Q20 (%)** | **Q30 (%)** | **GC content (%)** | **Total mapped** | **Multiple mapped** | **Uniquely mapped** |
| --- | --- | --- | --- | --- | --- | --- | --- | --- | --- | --- | --- | --- | --- | --- | --- | --- | --- | --- |
| **Skin** | Control | CKSK01 | 84.7 | 2.96 | 2.03 | 2.22 | 8.1 | 45223102 | 6828688402 | 43840666 | 6374714970 | 0.0254 | 97.83 | 93.96 | 47.44 | 32543301 (74.23%) | 1532939 (3.5%) | 31010362 (70.73%) |
|  |  | CKSK02 | 60 | 2.1 | 2.01 | 2.1 | 7.6 | 50791398 | 7669501098 | 49039582 | 7075292248 | 0.0259 | 97.63 | 93.41 | 48.09 | 37165802 (75.79%) | 2102208 (4.29%) | 35063594 (71.5%) |
|  |  | CKSK03 | 112.1 | 3.92 | 1.99 | 1.91 | 7.1 | 46271438 | 6986987138 | 44354292 | 6285536340 | 0.026 | 97.61 | 93.48 | 46.81 | 31977986 (72.1%) | 2009945 (4.53%) | 29968041 (67.57%) |
|  |  | CKSK04 | 147.9 | 5.18 | 2.01 | 2.28 | 7.6 | 50363544 | 7604895144 | 48552762 | 6890325734 | 0.0254 | 97.86 | 94.02 | 47.54 | 35534584 (73.19%) | 2198408 (4.53%) | 33336176 (68.66%) |
|  | *V. mimicus* | VMSK01 | 84.3 | 2.95 | 2.01 | 2.06 | 5.4 | 44093592 | 6658132392 | 43653198 | 6516564298 | 0.0267 | 97.43 | 92.55 | 46.56 | 14447609 (33.1%) | 793489 (1.82%) | 13654120 (31.28%) |
|  |  | VMSK02 | 103.7 | 3.63 | 2.03 | 2.18 | 7.9 | 48606604 | 7339597204 | 48186150 | 7146413235 | 0.028 | 96.9 | 91.42 | 46.44 | 34747093 (72.11%) | 1674293 (3.47%) | 33072800 (68.64%) |
|  |  | VMSK03 | 156.5 | 5.48 | 2.08 | 2.08 | 8 | 51056170 | 7709481670 | 50640408 | 7535114202 | 0.028 | 96.94 | 91.44 | 47.15 | 37034004 (73.13%) | 1935297 (3.82%) | 35098707 (69.31%) |
|  |  | VMSK04 | 182.9 | 6.4 | 2.01 | 2.33 | 7.3 | 42894632 | 6477089432 | 42452264 | 6253045447 | 0.0277 | 97.01 | 91.72 | 45.4 | 28036225 (66.04%) | 1707545 (4.02%) | 26328680 (62.02%) |
| **Muscle** | Control | CKMU01 | 160.9 | 5.63 | 1.99 | 2.05 | ＞8.0 | 49450016 | 7466952416 | 47684882 | 7020727632 | 0.0257 | 97.7 | 93.53 | 48.5 | 39960870 (83.8%) | 6481395 (13.59%) | 33479475 (70.21%) |
|  |  | CKMU02 | 91.2 | 3.19 | 2 | 1.89 | ＞8.0 | 53861012 | 8133012812 | 52658590 | 7752958591 | 0.0256 | 97.75 | 93.63 | 48.75 | 44436344 (84.39%) | 6952007 (13.2%) | 37484337 (71.18%) |
|  |  | CKMU03 | 104.2 | 3.65 | 2.02 | 1.85 | ＞8.0 | 70229368 | 10604634568 | 68240208 | 10033318609 | 0.026 | 97.59 | 93.26 | 48.65 | 56521352 (82.83%) | 7420528 (10.87%) | 49100824 (71.95%) |
|  |  | CKMU04 | 52.2 | 1.83 | 2 | 1.81 | ＞8.0 | 49335246 | 7449622146 | 47290222 | 7013970940 | 0.026 | 97.6 | 93.27 | 48.14 | 38596068 (81.62%) | 3352064 (7.09%) | 35244004 (74.53%) |
|  | *V. mimicus* | VMMU01 | 129 | 4.52 | 2.02 | 1.78 | ＞8.0 | 51576788 | 7788094988 | 50030176 | 7341569188 | 0.0257 | 97.7 | 93.53 | 48.88 | 40319060 (80.59%) | 3492612 (6.98%) | 36826448 (73.61%) |
|  |  | VMMU02 | 160.5 | 5.62 | 2.03 | 1.83 | ＞8.0 | 56848840 | 8584174840 | 55129210 | 8079497790 | 0.0259 | 97.66 | 93.42 | 48.63 | 44519895 (80.76%) | 5387354 (9.77%) | 39132541 (70.98%) |
|  |  | VMMU03 | 354.9 | 12.42 | 2 | 2.22 | ＞7.5 | 65326538 | 9864307238 | 63042166 | 9277290435 | 0.0267 | 97.28 | 92.71 | 46.77 | 45001030 (71.38%) | 2821673 (4.48%) | 42179357 (66.91%) |
|  |  | VMMU04 | 94 | 3.29 | 1.99 | 1.94 | ＞8.0 | 59802522 | 9030180822 | 57802288 | 8539900785 | 0.0257 | 97.73 | 93.59 | 48.89 | 48475942 (83.87%) | 6716928 (11.62%) | 41759014 (72.24%) |

**Supplementary Table 3**. Function annotation statistics

| Database | Transcriptome | | | | | | 4D Label-free | |
| --- | --- | --- | --- | --- | --- | --- | --- | --- |
|  | Skin | | Muscle | | Genome | | Skin | |
|  | Expre_Gene number (percent) | Expre_Transcript number (percent) | Expre_Gene number (percent) | Expre_Transcript number (percent) | All_Gene number (percent) | All_Transcript number (percent) | Protein Number | Percentage |
| GO | 19871 (0.7585) | 40140 (0.7614) | 19782 (0.7617) | 40094 (0.7634) | 22926 (0.6758) | 49057 (0.717) | 1705 | 0.7002 |
| KEGG | 18382 (0.7017) | 38915 (0.7382) | 18291 (0.7043) | 38811 (0.739) | 23131 (0.6819) | 49197 (0.719) | 1703 | 0.6994 |
| COG | 23337 (0.8908) | 48641 (0.9227) | 23198 (0.8932) | 48513 (0.9237) | 24804 (0.7312) | 57291 (0.8373) | 1865 | 0.7659 |
| Pfam | 21507 (0.821) | 44752 (0.8489) | 21419 (0.8247) | 44683 (0.8508) | 22473 (0.6625) | 52122 (0.7618) | 1832 | 0.7524 |
| NR | 25189 (0.9615) | 51573 (0.9783) | 24995 (0.9624) | 51405 (0.9788) | 31391 (0.9254) | 65561 (0.9582) | / | / |
| Swiss-Prot | 22474 (0.8579) | 47191 (0.8952) | 22352 (0.8607) | 47150 (0.8978) | 23701 (0.6987) | 55375 (0.8093) | / | / |
| SubCell-Location | / | / | / | / | / | / | 1886 | 0.7745 |
| Total_anno | 25198 (0.9619) | 51583 (0.9785) | 25006 (0.9628) | 51417 (0.979) | 31404 (0.9258) | 65575 (0.9584) | 1886 | 0.7745 |
| Total | 26197 (1.0) | 52717 (1.0) | 25971 (1.0) | 52518 (1.0) | 33922 (1) | 68423 (1) | 2435 | 1 |





**Supplementary Figure 1.** Data quality control of skin 4D-Label free analysis.

A: Distribution of peptide length. B: Information on identified proteins. C: Distribution of protein molecular weights. D: Distribution of protein coverage. E: Statistics on functional annotations.


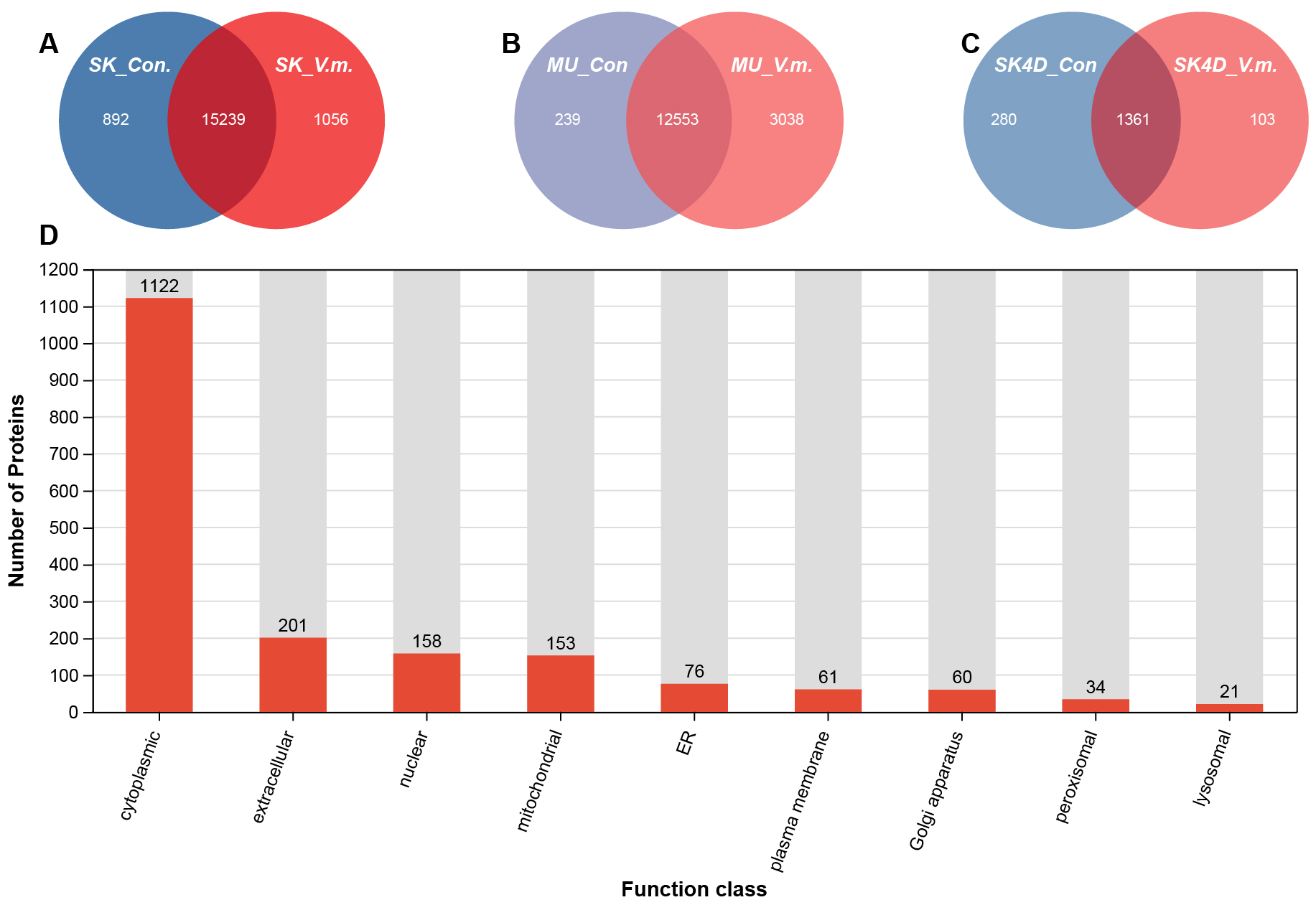


**Supplementary Figure 2.** Expression analysis of annotated genes and proteins in yellow catfish infected with *V. mimicus*

A: Venn diagram illustrating the gene annotation overlap between skin transcriptomes of the infected and control groups. B: Venn diagram illustrating the gene annotation overlap between muscle transcriptomes of the infected and control groups. C: Venn diagram illustrating the overlap of differentially expressed proteins identified in skin 4D Label-free analysis. D: Subcellular localization annotation of proteins identified in the infected and control groups.


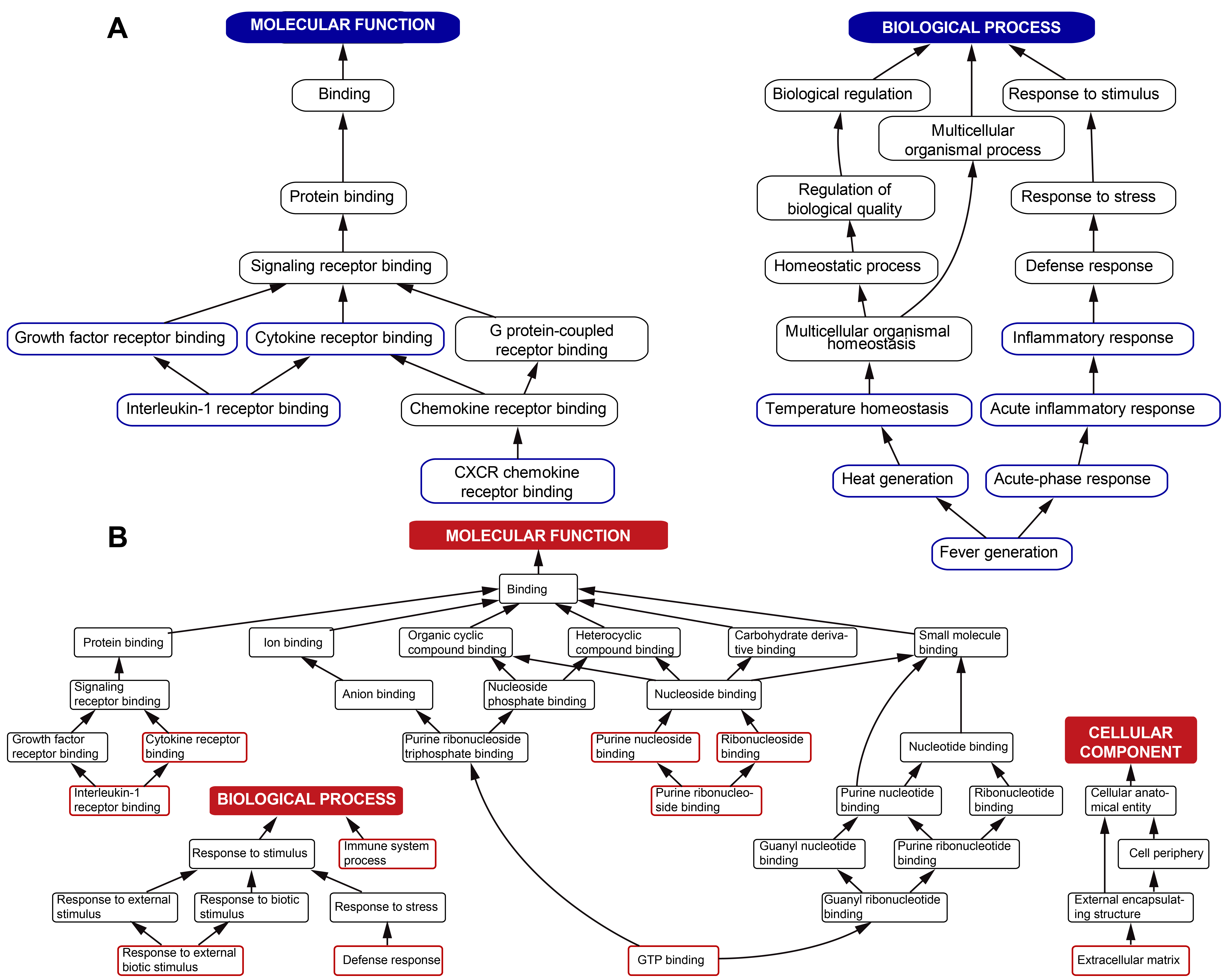


**Supplementary Figure 3.** The Directed Acyclic Graph (DAG) analysis of Gene Ontology (GO) enrichment for differentially expressed genes in the skin (Panel A) and muscle (Panel B).


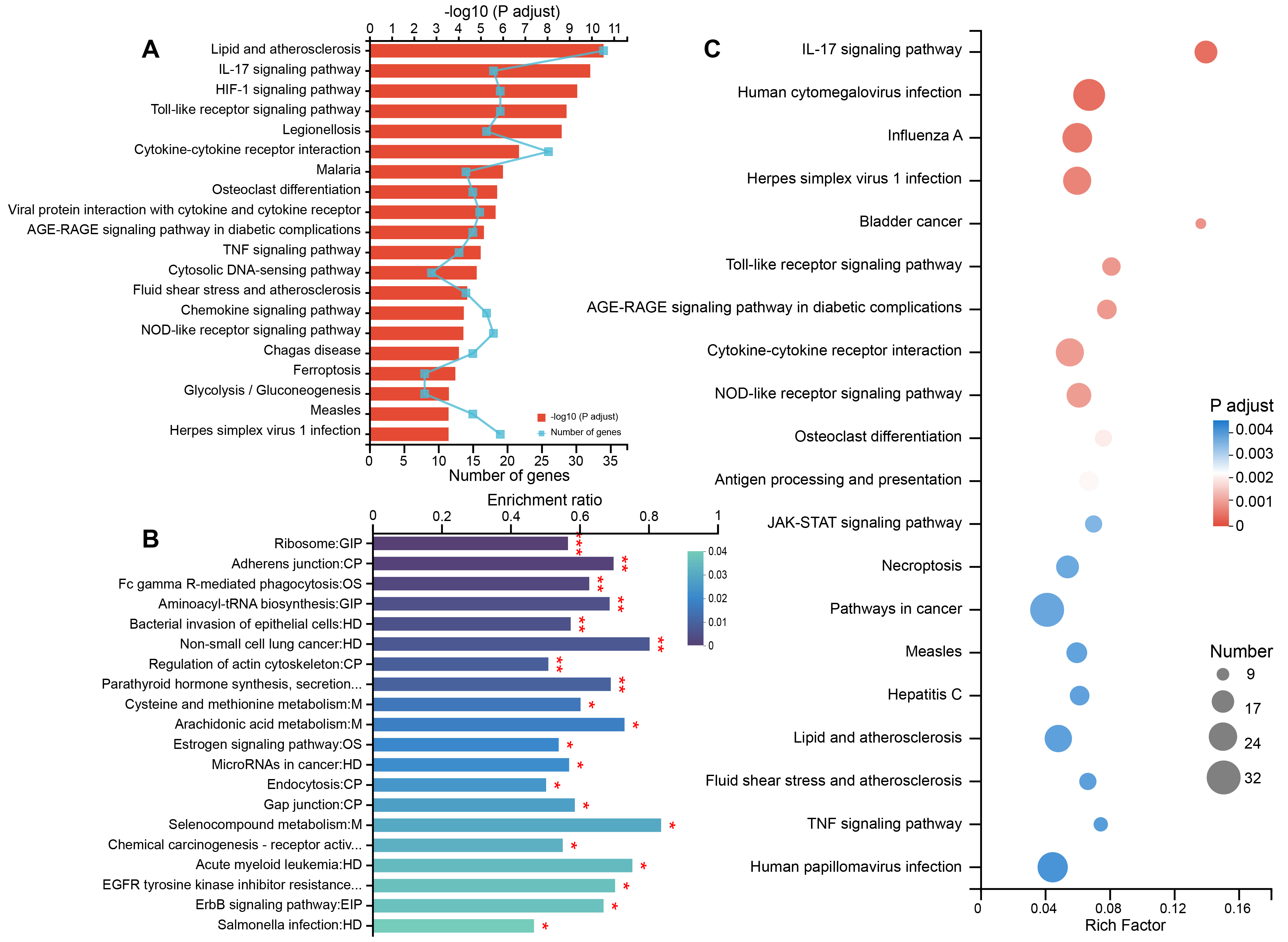


**Supplementary Figure 4.** The KEGG annotation analysis of genes.

A: The KEGG annotation analysis between the skin transcriptomes of the infected and control groups. B: The KEGG annotation analysis of differentially expressed proteins identified in the skin 4D Label-free analysis. C: The KEGG annotation analysis between the muscle transcriptomes of the infected and control groups.

**References**

Cheng, K., C. Ma, X. Guo, Y. Huang, R. Tang, N. A. Karrow and C. Wang, 2020. Vitamin D3 modulates yellow catfish (*Pelteobagrus fulvidraco*) immune function in vivo and in vitro and this involves the vitamin D3/VDR-type I interferon axis. Developmental & Comparative Immunology. 107, 103644.

Ke, F., Y. Wang, J. Hong, C. Xu, H. Chen and S.-B. Zhou, 2015. Characterization of MMP-9 gene from a normalized cDNA library of kidney tissue of yellow catfish (*Pelteobagrus fulvidraco*). Fish & Shellfish Immunology. 45(2), 260-267.

Li, D.-D., S.-C. Ling, K. Wu and Z. Luo, 2019. Identification of five key genes involved in intrinsic apoptotic pathway from Yellow Catfish *Pelteobagrus fulvidraco* and their transcriptional responses to high fat diet (HFD). Frontiers in Physiology. 10, 921.

Wang, H., S. Li, Z. Wu, L. Xu, D. Tian and X. Chen, 2020. Identification and functional gene expression analysis of Langerhans cells in yellow catfish (*Pelteobagrus fulvidraco*). Jorunal of Huazhong Agricultural University (in Chinese). 39(6), 173-179.

Zhang, M., X. Yin, M. Li, R. Wang, Y. Qian and M. Hong, 2020. Effect of nitrite exposure on haematological status, oxidative stress, immune response and apoptosis in yellow catfish (*Pelteobagrus fulvidraco*). Comparative Biochemistry and Physiology Part C: Toxicology & Pharmacology. 238, 108867.

Zhong, A. and T. Gao, 2022. Transcriptome analysis reveals similarities and differences in immune responses in the head and trunk kidneys of yellow catfish (*Pelteobagrus fulvidraco*) stimulated with Aeromonas hydrophila. Fish & Shellfish Immunology. 130, 155-163.

Zhou, X., G.-R. Zhang, W. Ji, Z.-C. Shi, X.-F. Ma, Z.-L. Luo and K.-J. Wei, 2021. The dynamic immune response of yellow catfish (*Pelteobagrus fulvidraco*) infected with Edwardsiella ictaluri presenting the inflammation process. Frontiers in Immunology. 12, 625928.

Zhou, X., G.-R. Zhang, W. Ji, Z.-C. Shi, X.-F. Ma, Z.-L. Luo and K.-J. Wei, 2021. Expression and function analysis of interleukin-17A/F1, 2, and 3 genes in yellow catfish (*Pelteobagrus fulvidraco*): distinct bioactivity of recombinant IL-17A/F1, 2, and 3. Frontiers in Immunology. 12, 626895.
